# Advances in Management of Mitochondrial Myopathies

**DOI:** 10.3390/ijms26115411

**Published:** 2025-06-05

**Authors:** Athanasios Bangeas, Vasiliki Poulidou, Ioannis Liampas, Chrysa Marogianni, Athina-Maria Aloizou, Zisis Tsouris, Markos Sgantzos, Marianthi Arnaoutoglou, Dimitrios P. Bogdanos, Efthimios Dardiotis, Vasileios Siokas

**Affiliations:** 1 Department of Neurology, Laboratory of Neurogenetics, University Hospital of Larissa, Faculty of Medicine, School of Health Sciences, University of Thessaly, 41100 Larissa, Greece; th.mpagg@gmail.com (A.B.); liampasioannes@gmail.com (I.L.); c.marogianni@gmail.com (C.M.); athena_aloi@yahoo.gr (A.-M.A.); tsouriszisis@me.com (Z.T.); sgantzos@med.uth.gr (M.S.); edar@med.uth.gr (E.D.); 2 First Department of Neurology, AHEPA University Hospital, Aristotle University of Thessaloniki, Stilponos Kyriakidi 1, 54636 Thessaloniki, Greece; basia_poulidou@yahoo.gr; 3 Neurology Department, St. Josef Hospital Bochum, Ruhr University Bochum, Gudrunstr. 56, 44791 Bochum, Germany; 4 Department of Clinical Neurophysiology, School of Medicine, AHEPA University Hospital, Aristotle University of Thessaloniki, Stilponos Kyriakidi 1, 54636 Thessaloniki, Greece; marnaoutoglou@yahoo.com; 5 Department of Rheumatology and Clinical Immunology, University General Hospital of Larissa, Faculty of Medicine, School of Health Sciences, University of Thessaly, 41100 Larissa, Greece; bogdanos@uth.gr

**Keywords:** mitochondrial myopathies, treatment, management

## Abstract

Mitochondria, the energy factories of human organisms, can be the cause of a variety of genetic disorders called mitochondrial myopathies. Mitochondrial diseases arise from genetic alterations in either mitochondrial DNA (mtDNA) or nuclear DNA (nDNA) and can manifest with great heterogeneity, leading to multiorgan dysfunction. The purpose of this article is to concisely review the pathophysiology, genetics and main clinical features of mitochondrial myopathies, focusing mainly on the treatment and management of these disorders. Currently, a particular treatment for mitochondrial myopathies does not exist, while the available guidelines concerning management are based on experts’ opinions. The therapeutic options currently applied largely aim at symptom relief and amelioration of patients’ quality of life. The most commonly used regimens involve the administration of vitamins and cofactors, although hard evidence regarding their true benefit for patients is still lacking. Recent studies have demonstrated promising results for elamipretide; however, phase III clinical trials are still ongoing. Regarding patient management, a multidisciplinary approach with the collaboration of different specialties is required. Further clinical trials for the already applied treatment options, as well as on novel experimental therapies, are of utmost importance in order to improve patients’ outcomes.

## 1. Introduction

Mitochondrial myopathies represent a broad and diverse group of genetic disorders, exhibiting a wide range of phenotypic manifestations. They are part of the super family of mitochondrial diseases, which derive from a primary dysfunction of the mitochondrial respiratory chain, leading mostly to muscle disorders, hence the term mitochondrial myopathies. Apart from skeletal muscle cells, other organs e.g., the brain and liver, have high energy demands, which are mainly met by mitochondrial energy production. As a result, these disorders are characterized not only by muscle disease but also by dysfunction in various organ systems, with clinical manifestations that often show poor correlation with the underlying genetic alterations. This lack of straightforward genotype-phenotype correlation can be attributed to several factors. First, unlike the nuclear genome, which is diploid, the mitochondrial genome is polyploid, with mitochondrial DNA (mtDNA) copy number varying significantly between different cell types and tissues-often reaching hundreds to thousands of copies per cell, depending on cellular energy demand [1]. This variability of copy number significantly impacts the phenotypic expression of a mutation. Second, the phenomenon of heteroplasmy, which refers to the proportion of wild-type (normal) and mutated mtDNA that are contained within a cell, results in variable expression of disease depending on the mutation load across different tissues [2]. Third, the threshold effect, where a critical proportion (80–90%) of mutated mtDNA must be exceeded before mitochondrial diseases manifest, further complicates the genotype-phenotype relationship [3]. Finally, nuclear genetic background and environmental factors can modulate the severity and features of mitochondrial diseases, adding another layer of variability [4]. To better understand this variability, it is essential to examine both the pathophysiological mechanisms of mitochondrial dysfunction and the genetic determinants that shape disease expression.

### 1.1. Pathophysiology

Mitochondria are intracellular organelles that play a key role in energy production and, in a manner of speaking, represent the ‘power factories’ of all mammalian organisms. Approximately 90% of the energy needs of the body’s tissues are met by them [5]. Mitochondria generate energy in the form of ATP via the respiratory chain. The respiratory chain, also known as the electron transport chain, is made of four multicomplex proteins, embedded within the inner mitochondrial membrane.

Complex I, also known as nicotinamide adenine dinucleotide (NADH) dehydrogonase or NADH quinone reductase, is the largest complex of the respiratory chain. It is composed of 45 subunits, seven of which are encoded by mtDNA and 38 of which are ecnoded by nuclear DNA (nDNA). Complex I allows the electron transfer, which leads to changes in the structure of complex I subunits and finally to proton passage from the matrix side to the inner-membrane space [6]. Complex I deficiency is associated with Leigh’s disease and Leber’s hereditary optic neuropathy (LHON). Complex II, or succinate coenzyme Q reductase, is the only complex of the respiratory chain, the four subunits of which are entirely encoded by nDNA. The role of this complex lies in the transfer of electrons from the citric acid cycle to the oxidative phosphorylation (OxPhos) electron carrier ubiquinone. Although complex II deficiency is rare, mutations in the *SDH-A* gene can cause Leigh-like syndromes [7]. Ubiquinone-cytochrome c oxidoreductase or complex III consists of 11 subunits. All of these subunits are encoded by nDNA except cytochrome b, which is regulated by mtDNA. The main function of this complex is the electron transfer to cytochrome c, which is linked to the production of reactive oxygen species (ROS) [8]. The function of complex IV or cytochrome oxidase c (COX) is related to electron transfer, which is regulated by the three large core subunits (COX1, COX2, COX3) that are mtDNA encoded. The other 10 nDNA encoded subunits are important for the stabilization of the complex’s structure [9]. Mitochondrial encephalomyopathy lactic acidosis and stroke-like episodes (MELAS syndrome) is related with mutations of COX genes in the mtDNA. Finally, complex V or ATP synthase consists of two parts, F_0_ and F_1_, and regulates ADP phosphorylation. ATP6, ATP8 and two F_0_ subunits are mtDNA encoded, whereas all of the F_1_ subunits are nDNA encoded [10].

**Figure 1:**
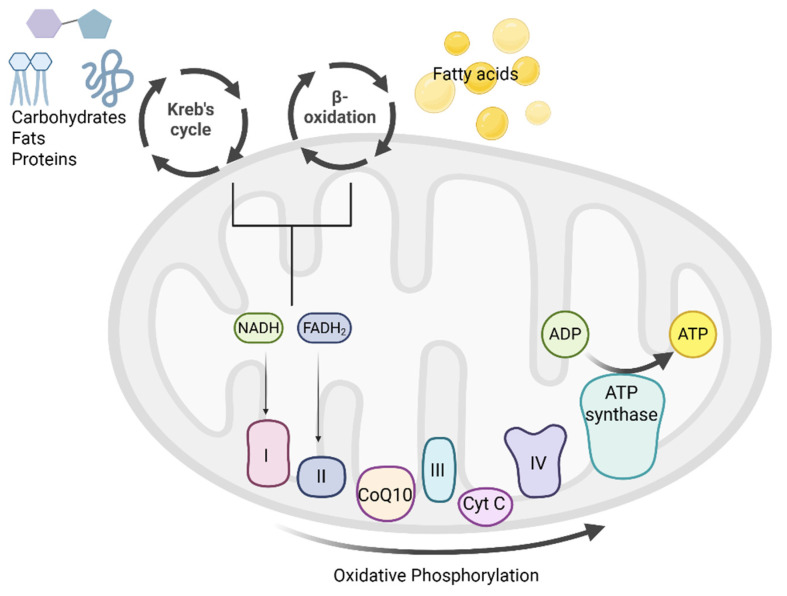
The physiology behind ATP formation. Within the mitochondrion, carbohydrates, fats, proteins and fatty acids are metabolized through the citric acid cycle and β-oxidation, producing NADH and FADH₂. These electron donors fuel the respiratory chain, which comprises four multi-subunit complexes (I–IV), via electron carriers (CoQ10 and cytochrome c), driving proton translocation across the inner mitochondrial membrane. The resulting electrochemical gradient drives ATP synthesis by complex V (ATP synthase), which catalyzes the phosphorylation of ADP to ATP. ADP: adenosine diphosphate, ATP: adenosine triphosphate, CoQ10: coenzyme Q10, Cyt C: cytochrome c, FADH2: flavin adenine dinucleotide hydrogenated, NADH: nicotinamide adenine dinucleotide. The figure was prepared using the BioRender platform (under license to DPB, Created in BioRender. Bogdanos, D. (2025) https://BioRender.com/6i718nb).

Inside the mitochondrion, carbohydrates, fats and proteins are metabolized via the citric acid cycle (Krebs cycle), while fatty acids are metabolized via the β-oxidation cycle. These processes result in the production of NADH and flavin adenine dinucleotide hydrogenated (FADH_2_), which donate electrons to the complexes of the respiratory chain with the aid of electron carriers (coenzyme Q10 and cytochrome c). As a consequence, protons are translocated across the inner mitochondrial membrane space, and this proton accumulation comprises the main force of ATP formation. The formation of ATP results from the condensation of ADP and a phosphate, in the form of PO^−^_4_, which is known as OxPhos and takes place within complex V of the respiratory chain [11,12,13] (Figure 1).

### 1.2. Genetics

Mitochondria contain their own genetic material, mtDNA. However, the mitochondrial function is also under the control of the nDNA, which regulates the maintenance of mtDNA, the mitochondrial protein synthesis and the synthesis and function of the respiratory chain complexes and co-factors [14,15]. Mitochondrial diseases and myopathies are caused by mutations to either the mtDNA or the nDNA [16]. A large number of mutations in mtDNA and nDNA genes have been linked to mitochondrial myopathies and diseases in general, each through distinct mechanisms [17]. MtDNA mutations directly affect mitochondrial function by disrupting essential components of the respiratory chain and affecting protein synthesis. For example, mutations in the MT-TL1 gene, which encodes mitochondrial tRNA, or in the MT-ATP6 gene, encoding a subunit of ATP synthase, are linked to disorders such as MELAS and neuropathy, ataxia, and retinitis pigmentosa syndrome (NARP) [18]. In contrast, nDNA mutations affect the function of the mitochondrial respiratory chain either directly—such as through mutations in genes encoding respiratory chain subunits or indirectly—such as through mutations in genes encoding assembly proteins [18]. Additionally, mutations in nDNA genes can affect mtDNA replication, repair or maintenance [18]. Mutations in genes like POLG (mitochondrial DNA polymerase) or OPA1 (involved in mitochondrial fusion) are examples of nuclear mutations that affect mitochondrial function [15,18]. Mitochondrial DNA is maternally inherited; therefore, mtDNA mutations are passed down from the mother to all their children. It should be noted, though, that some mtDNA mutations can occur during embryonic development [19]. Primary mtDNA mutations encompass point mutations, single large-scale deletions, mtDNA depletion and multiple deletions. Single large-scale deletions are typically sporadic and not inherited, whereas mtDNA depletion and multiple deletions arise secondarily from mutations in nDNA, which encodes mitochondrial proteins. Nuclear DNA mutations follow the Mendelian rules and, as such, are either autosomal dominant, autosomal recessive or X-linked. On the other hand, the inheritance of mtDNA mutations is correlated to a certain level of heteroplasmy, which is unpredictable and quite random. This is the reason why even among family members, the severity of mitochondrial disease is variable. With the identification of new genes in mitochondrial disease at a rapid pace in recent years, the diagnosis of these diseases is starting to shift towards molecular genetics studies, including next-generation sequencing (NGS) techniques [20].

### 1.3. Epidemiology

Estimating the exact frequency of mitochondrial diseases is a quite challenging task, as they represent disorders with great phenotypic and genetic heterogeneity. They seem to affect 1:4300 individuals [14,15], although carriers of mitochondrial genetic defects are estimated to reach rates of 1 in 200–250 individuals [21,22]. Data from the United States suggest an annual rate of 1000–4000 births with a mitochondrial disorder [23]. Moreover, the prevalence of mitochondrial diseases appears to vary between different geographic populations, as suggested by studies in Finland [24].

### 1.4. Clinical Manifestations

The age of onset and severity of clinical features, as well as specific organ involvement, are highly variable in mitochondrial disorders, especially in those stemming from mutations in mtDNA, due to heteroplasmy [25]; the higher the percentage of mutant heteroplasmy, the younger the onset of age and the higher the severity of the disease.

As mentioned above, mitochondria are considered the ‘power-plants’ in mammalian cells, meaning that the clinical manifestation of mitochondrial disease affects tissues and organs that have high energy demands. The most common and prevalent feature of almost all mitochondrial disorders is myopathy, as skeletal and smooth muscles are rich in mitochondria [18,26]. Mitochondrial myopathies may present with progressive muscle weakness, easy and early fatigue, and exertional intolerance. In these patients, limitations in exercise capacity can also occur due to a low VO_2max_ (maximal oxygen consumption) [27]. In general, mitochondrial myopathies tend to be symmetric and initially affect the more proximal muscles, and, alongside skeletal muscles, smooth muscle and cardiac muscle can be affected too. Extraocular muscles are most often and prematurely affected in mitochondrial myopathies, leading to ptosis and ophthalmoparesis, with progressive external ophthalmoplegia (PEO) being a very important sign [28]. Mitochondrial myopathies can also cause muscle weakness, dystrophy or atrophy in other facial and neck muscles, which may impede speech and even swallowing. Involvement of the muscles that support breathing may lead to the need for ventilator-supported breathing in these patients. The myopathy can manifest at any age; however, it seems to more often begin at an older age [25]. Symptoms of weakness, exercise intolerance and fatigue are frequently not mentioned in the very first clinical evaluation and may be underestimated but can be brought to light in combination with symptoms from other organs and systems.

Since skeletal muscles are the most commonly affected system in mitochondrial diseases, muscle biopsy remains a crucial diagnostic tool for these disorders. Patients with mitochondrial myopathies appear to have histochemical changes in their skeletal muscles, although that is not always the case, as sometimes muscle biopsy may appear normal [17]. Defects in OxPhos function can be detected as the presence of ragged-red fibers (RRF) in skeletal muscle biopsy via staining with the modified Gomori trichrome or using succinate dehydrogenase (SDH, complex II) histochemistry [29]. Another histological characteristic of mitochondrial myopathies is the presence of cytochrome c oxidase (COX, complex IV)—negative fibers which are stained blue and give the appearance of a mosaic pattern with the normal COX-positive fibers, which are stained brown [30]. Other features in muscle biopsy which are non-specific include atrophy, internal nuclei, lipid or glycogen accumulation and fiber size variations [17].

Other tissues with high energy requirements, beside muscles, are the nervous system, heart, liver, kidneys and endocrine system, translating to a variety of clinical manifestations and multisystem dysfunction. The nervous system manifestations include ataxia, migraine, seizures, strokes and stroke-like episodes (as described in MELAS syndrome), deafness, peripheral neuropathy, developmental delay and even neuropsychiatric symptoms such as depression and psychosis. EEG findings may be indicative of subclinical epilepsy, whereas findings in MRI, such as restricted diffusion or (FLAIR)/T_2_ changes consistent with stroke can be found in patients with MELAS syndrome [17,18,31].

The involvement of the cardiac muscle in mitochondrial myopathies is possibly a result of cardiac myopathy as well, although it is not a frequent complication of mitochondrial dysfunction. Patients may present with conduction abnormalities and arrhythmias and hypertrophic or dilated cardiomyopathy, which can even result in heart failure [32,33].

Liver dysfunction and liver failure are most common in infants with mtDNA depletion disorders, while renal dysfunction in mitochondrial diseases is mostly associated with proximal tubulopathy [34,35,36,37]. Gastrointestinal symptoms usually manifest as pseudo-obstruction and dysmotility due to nerve or smooth muscle involvement [38]. Endocrine system dysfunction in mitochondrial diseases typically includes diabetes mellitus, especially in patients with MELAS, whilst cases of hypoparathyroidism, adrenal insufficiency and thyroid function disorders have also been described in literature [39,40,41,42,43].

**Figure 2:**
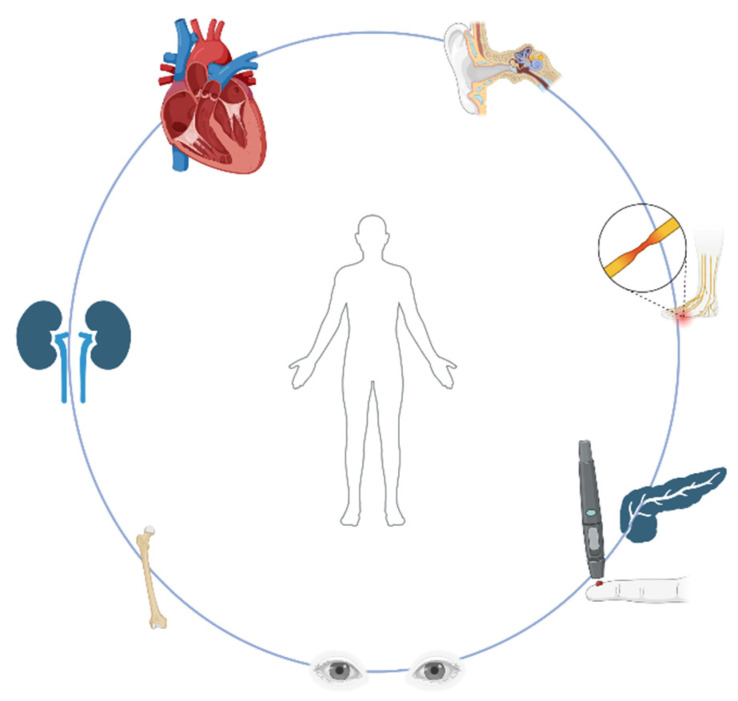
The red flags for mitochondrial diseases. These clinical indicators should raise suspicion for mitochondrial myopathies in pediatric patients and prompt clinicians to pursue further diagnostic evaluation. Key signs include neurosensory hearing loss, short stature, ophthalmoplegia, ptosis, neuropathy, renal tubular acidosis, diabetes and hypertrophic cardiomyopathy. The figure was prepared using the BioRender platform (under license to DPB, created in BioRender. Bogdanos, D. (2025) https://BioRender.com/bqcpibw).

As far as children are concerned, developmental delays including delays in achieving specific milestones by age in motor, language and learning skills, which may be due to muscle weakness or brain abnormalities, may prompt pediatricians to further investigate for mitochondrial myopathies [28]. The so-called ‘red flag signs’ (Figure 2), which should alarm every clinician, include neurosensory hearing loss, short stature, ophthalmoplegia, ptosis, neuropathy or nerve disease, renal tubular acidosis, diabetes and hypertrophic cardiomyopathy [42].

#### Clinical Syndromes

**Figure 3:**
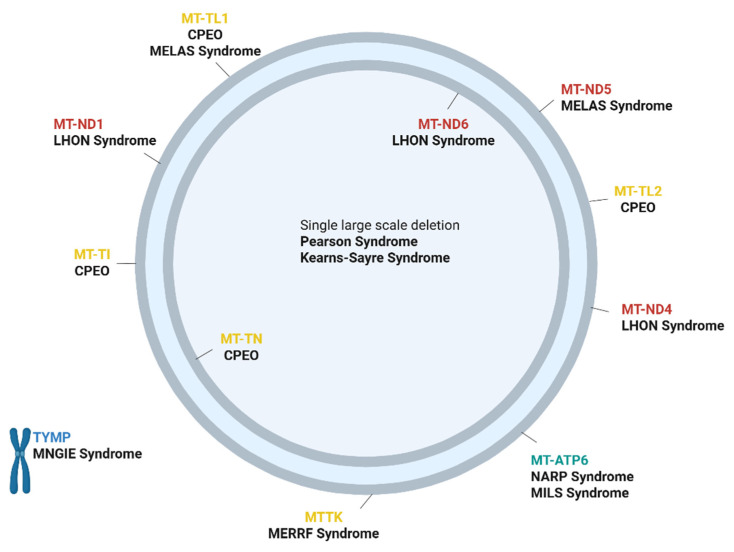
Main genetics of primary mitochondrial myopathy syndromes. This figure highlights the most common genetic factors associated with primary mitochondrial myopathy syndromes. Complex I genes are indicated in red, complex V genes are in green, tRNA genes are in yellow and nuclear gene is in blue. CPEO: chronic progressive external ophthalmoplegia: m.3243A>G, m.3243A>T, m.4298G>A, m.4308G>A, m.5690A>G, m.5703G>A, m.12276G>A, m.12294G>A, m.12315G>A, m.12316G>A (MT-TL1, MT-TI, MT-TN, MT-TL2 genes), LHON: Leber’s hereditary optic neuropathy: m.3460G>A (MTND1 gene), m.14484T>C (MTND6 gene), m.11778G>A (MTND4 gene), MELAS: mitochondrial encephalomyopathy lactic acidosis and stroke-like episodes: m.3243A>G (MTTL gene), m.13513G>A (MTND5 gene), MERF: myoclonus, epilepsy and red ragged fibers: m.8344A>G (MTTK gene), MILS: maternally inherited Leigh syndrome: 90% mutation in MT-ATP6 gene, MNGIE: mitochondrial neurogastrointestinal encephalopathy: mutations in TYMP gene, NARP: neuropathy, ataxia and retinitis pigmentosa: 70% mutation in MT-ATP6 gene [20,44,45,46,47,48,49,50,51]. The figure was prepared using the BioRender platform (under license to DPB, created in BioRender. Bogdanos, D. (2025) https://BioRender.com/hazngl0).

As mentioned earlier, mitochondrial myopathies can manifest with great heterogeneity. Classical mitochondrial syndromes in which myopathy is a common clinical feature are well described in both adult and children populations in literature (Figure 3).

Ocular muscle weakness is one of the most common features of mitochondrial myopathies. Chronic progressive external ophthalmoplegia (CPEO) is characterized by symmetric or asymmetric slowly progressive ophthalmoplegia and ptosis and can manifest at any age of onset. Patients may also develop oropharyngeal and proximal weakness. CPEO may be sporadic, maternally inherited, autosomal dominant or recessive [43,52]. Among the nuclear genes associated with autosomal dominant and autosomal recessive forms of CPEO, POLG is the most frequently implicated, with other commonly involved genes including WFS1, C10orf2, SLC25A4, RRM2B, POLG2 and SPG7 [53]. PEO can also be part of the presentation of ataxia-neuropathy syndromes (ANS) [54].

MELAS is a complex syndrome presenting with multi-organ dysfunction with onset usually before 40 years of age. Central nervous system dysfunction predominates, and the syndrome is characterized by stroke-like episodes, encephalopathy with headaches, seizures, cognitive impairment, peripheral neuropathy, gastrointestinal symptoms, lactic acidosis and diabetes, besides myopathy [55]. In infants, it may present with psychomotor developmental delay and learning disability. The initial diagnostic criteria for MELAS included stroke-like episodes occurring before the age of 40, encephalopathy characterized by seizures or dementia and the presence of either blood lactic acidosis or ragged red fibers in skeletal muscle biopsy [56]. Recently, the MELAS Study Group Committee in Japan introduced updated diagnostic criteria. Under these new guidelines, a definitive diagnosis of MELAS requires the presence of at least two category A criteria (which include headaches with vomiting, seizures, hemiplegia, cortical blindness and acute focal lesions detected through neuroimaging) along with two category B criteria (such as elevated lactate levels in plasma or cerebrospinal fluid, mitochondrial abnormalities observed in muscle biopsy and identification of a MELAS-associated gene mutation) [57]. MELAS is maternally inherited, with the m.3243A>G mutation in the MT-TL1 gene present in over 80% of cases, while other mtDNA variants, including m.3271T>C and m.3252A>G in MT-TL1, can also contribute to the condition [58,59].

Kearns–Sayre syndrome (KSS) represents a distinct subtype of CPEO characterized by the diagnostic triad of CPEO, pigmentary retinopathy and disease onset before the age of 20. Additionally, at least one of the following features must be observed: cardiac conduction abnormalities, cerebellar ataxia or an elevated cerebrospinal fluid protein level (greater than 100 mg/dL) [60]. Other clinical features that may be observed in KSS include short stature, anemia, diabetes, hearing loss and cognitive impairment. It is either sporadic or maternally inherited and is characterized by variable single mtDNA deletions [43,60].

LHON is a painless, bilateral, subacute optic neuropathy that predominantly affects males and leads to severe and permanent visual loss. The increased susceptibility in males lacks a clear genetic explanation; nevertheless, it is believed that the underlying pathogenic mechanism involves a severe impairment of complex I-dependent ATP synthesis [61]. The diagnosis is usually confirmed through the identification of pathogenic variants via genetic testing. Three mtDNA pathologic variants, at nucleotide positions 3460G>A in the gene ND1, 11778G>A in the gene ND4 and 14484T>C in the gene ND6, are specific for LHON and account for more than 90 percent of cases [62].

The myoclonus, epilepsy and red ragged fibers (MERF) syndrome is defined by myoclonus and generalized epilepsy, muscle weakness and progressive ataxia. MERF can be sporadic or maternally inherited and usually manifests during infancy. It is also associated with short stature, optic atrophy, hearing loss and Wolf–Parkinson–White syndrome [63]. In MERRF, several mtDNA mutations have been identified, with the most prevalent being an A-to-G substitution at nucleotide 8344 (m.8344A>G) in the mtDNA lysine tRNA gene (MT-TK), which is found in approximately 80% of affected individuals [64].

Mitochondrial neurogastrointestinal encephalopathy (MNGIE) represents a rare entity that is characterized by gastrointestinal dysmotility, cachexia, ophthalmoplegia, polyneuropathy and leukoencephalopathy. Cardinal tests to diagnose this disorder include swallowing assessment, gastric emptying studies and gastrointestinal manometry, brain MRI and nerve conduction studies [48]. The long-term prognosis of MNGIE remains poor. MNGIE is caused by pathogenic variants of the thymidine phosphorylase (TYMP) gene [48]. The subsequent accumulation of the toxic substrate thymidine in the plasma disrupts the nucleotide pool balance and results in mtDNA abnormalities [47].

Autosomal recessive mutations in the thymidine kinase 2 (TK2) gene can lead to damage in the maintenance and replication of mtDNA, causing TK2 deficiency. Three forms of the disease are described, namely the infantile, childhood and late-onset disease. In the first and most severe form, TK2 deficiency presents during infancy and is related to severe myopathy and early death. In the childhood-onset form, the disease usually manifests between 1 to 12 years of age and is characterized by proximal limb weakness with important respiratory insufficiency. The late-onset form presents at 12 years of age or older, and its clinical manifestations include PEO, dysphagia, dysarthria and myopathy. The involvement of respiratory muscles varies [65,66].

Coenzyme Q10 (CoQ10) biosynthesis depends on the activity of at least 13 genes [67]. Mutations in any of these genes can lead to primary CoQ10 deficiency, a disorder characterized by a variety of symptoms and organ involvement including muscle weakness, central nervous system involvement and peripheral neuropathy, steroid-resistant nephrotic syndrome, hypertrophic myocardiopathy and retinopathy. Although plasma CoQ10 levels can be measured, they often fail to reliably reflect tissue levels, making skeletal muscle, skin fibroblasts or CSF more appropriate for accurate assessment [68]. CoQ10 deficiency is crucial to recognize because it is a potentially treatable condition. Recent studies have shown that patients may benefit from high doses of oral CoQ10 supplementation [69].

## 2. Treatment and Management

As of now, no approved therapeutic agents or medications for mitochondrial myopathies exist that can either repair mitochondrial function or at least alter the course of the disease. Most therapies focus on symptomatic relief and management of disease manifestations, in order to improve the quality of life of these patients. These therapies include supplements like vitamins and cofactors, agents that may alter mitochondria function and biogenesis, exercise and diet options (Table 1).

However, the lack of evidence-based effectiveness even for these relief options necessitates a greater number of clinical trials and the need for approved treatment guidelines, as in most cases, even at present, the management and treatment of these patients is based upon experts’ opinions.

### 2.1. Dietary Interventions and Non-Specific Treatments

Dietary supplements often prescribed in combinations and characterized as ‘mitococktails’ are most frequently used in the treatment of primary mitochondrial myopathies (PMM), although evidence regarding the effectiveness of these therapies is still lacking [83]. These cocktails often consist of complex B vitamins, creatinine, coenzyme Q10, alpha lipoic and folinic acid and vitamins E and C. A Cochrane-based analysis involving 12 studies regarding the use of these factors in the treatment of mitochondrial myopathies led to controversial results and highlighted the need for further research [71]. Despite this fact, experts continue to recommend supplementation with vitamins and cofactors, since it has been considered a main pillar in the treatment of patients with mitochondrial myopathies for many decades now.

The most commonly recommended supplement is ubiquinol, a reduced form of CoQ10, which seems to improve symptoms especially in patients with primary Q10 deficiencies when provided in high doses (adults: p.o. 5–600 mg/daily, pediatric: p.o. 2–8 mg/kg/daily divided in two doses) [72,84].

Alpha lipoic acid and riboflavin (vitamin B2) are also being administered in most patients in doses 50–200 mg/daily and 50–400 mg/daily, respectively [18].

Nicotinamide riboside (NR), a derivative of vitamin B3, is a key precursor to NAD+ and can increase energy production and ATP levels by inducing mitochondrial biogenesis [73,85]. Studies in mice models have shown that the administration of NR may result in improvement of respiratory chain defects and a decrease in the accumulation of mtDNA deletions in skeletal muscles, thus preventing the progression of mitochondrial myopathies [86,87]. However, in spite of NR being a promising treatment for mitochondrial myopathies, hard evidence of its benefits in human patients is still lacking. A phase II randomized, placebo-controlled clinical trial evaluating the efficacy of high dose NR (1000 mg/day) for the treatment of mitochondrial myopathy is still ongoing (NCT05590468).

Carnitine is found to increase ATP production by mobilizing the use of fatty acids [88]. In the past, carnitine in the form of L-carnitine was widely used in these patients; however, it is no longer recommended as a routine treatment option unless there is a carnitine deficiency [25]. The same applies to folinic acid, which is prescribed by most experts in cases of cerebrospinal fluid folate deficiency, a quite common state in mtDNA depletion disorders [25].

Creatinine, as a reserve of ATP, can also be administered in patients with PMM, in a dose of 10 g p.o. daily divided two to three times and 0.1g/kg p.o. daily in children [71].

The use of both intravenous and oral L-arginine has been recommended in the acute treatment of stroke-like episodes in MELAS syndrome, but studies have also described its benefits in the amelioration of four major symptoms, including headache, nausea/vomiting, impaired consciousness and visual disturbance, in both adult and pediatric patients [88]. However, a recent systematic review highlighted the limited efficacy of L-arginine as both acute and preventive treatment for MELAS syndrome, emphasizing the lack of placebo-controlled randomized clinical trials [89].

In a phase III trial, high-dose oral taurine supplementation, a sulfur-containing β-amino acid, significantly reduced the recurrence of stroke-like episodes in patients with MELAS, with 60% achieving complete prevention and 80% showing ≥50% reduction in episode frequency. Taurine also increased the taurine modification of mitochondrial tRNA^Leu(UUR)^ from peripheral blood leukocytes, suggesting a potential disease-modifying effect [80].

Exercise training, including aerobic, endurance or resistance exercise, still remains one of the therapeutic options in patients with PMM, improving the patient’s strength, work capacity, fatigue and, overall, their quality of life [90,91,92,93,94,95,96,97]. Exercise has been found to stimulate mitochondrial biogenesis and reduce the proportion of mutated mtDNA in skeletal muscles. However, its role in the pathogenesis or the progression of disease is yet to be determined.

Ketogenic diets with low glucose and high fat content may be considered as a possible treatment option for mitochondrial diseases. Studies on mice models with mitochondrial myopathies in which the ketogenic diet was used have shown a reduction in the amount of cytochrome c oxidase negative muscle fibers, prevention of mitochondrial abnormalities and an increase in mitochondrial biogenesis, suggesting a slowdown in the progression of mitochondrial myopathy [98]. In a pilot study of five patients, a modified Atkins diet was applied, resulting in a subacute response after 1.5–2 weeks of diet, while in the 2-year follow up period, muscle strength was notably improved, suggesting activation of muscle regeneration [99]. Currently, a small number of papers (mostly case reports) in literature regarding the use of the ketogenic diet in mitochondrial disease patients exist, suggesting seizure control and improved muscular symptoms after its use [100]. Nonetheless, the need for further studying regarding the efficacy and safety of the ketogenic diet in these patients is crucial.

### 2.2. Potentially Disease-Modifying Treatments and Therapies Under Investigation

Idebenone, which is a synthetic analog of CoQ10 approved in Europe for the management of visual impairment in LHON, was initially tested in a randomized placebo-controlled trial of LHON patients [101]. Idebenone enhances cellular energy production and facilitates functional recovery of retinal ganglion cells by bypassing the impaired mitochondrial complex I. This process not only prevents further deterioration of vision but also promotes the recovery of visual function [102]. While the primary endpoint for optimal recovery in visual acuity did not reach statistical significance, a subgroup of patients with discordant visual acuities between eyes showed the greatest potential benefit from this safe and well-tolerated treatment [101]. The results of another study suggest that idebenone has a positive effect on improving visual function even in patients with established optic atrophy [103]. This response to treatment in chronic LHON could result from the reactivation of signal transduction in surviving dysfunctional retinal ganglion cells [103]. Two more studies conducted on Asiatic populations further supported the potential therapeutic effects of idebenone, as well as its favorable safety profile [74,104].

Gene therapy through the delivery of a wild-type version of the defective gene via a viral vector represents a novel therapeutic approach for LHON and has demonstrated promising outcomes in recent studies [105]. At present, the delivery of the wild-type MT-ND4 gene into the retinal ganglion cell nuclei enables the production of functional complex I subunits, which can then be incorporated into the mitochondrial respiratory chain. This method has been tested in several clinical trials, showing significant potential as a therapeutic strategy for LHON [106,107,108,109].

Two different management strategies apply to MNGIE. The first approach aims to reduce the circulating toxic levels of thymidine through peritoneal dialysis. The second strategy includes several therapeutic options, such as platelet infusions, allogeneic hematopoietic stem cell transplantation, enzyme replacement therapy using erythrocyte encapsulated thymidine phosphorylase and orthotopic liver transplantation, all of which aim to restore thymidine phosphorylase activity [110]. Enzyme replacement therapy for MNGIE has been successful in four patients described in the literature [111,112].

In recent years, a focused treatment for TK2 disease using deoxynucleoside monophosphates and deoxynucleosides showed promising results in mouse models, resulting in improvements in mtDNA depletion and OxPhos defects by bypassing the deficiency of the TK2 enzyme [113,114]. An open-label study of 16 patients revealed improved survival in early onset disease, as well as motor functions, with a favorable side effect profile [115], leading to further clinical trials for the approval of this treatment. An ongoing phase II clinical study (NCT04802707) aims to enroll 50 pediatric patients with mtDNA depletion syndromes to evaluate the safety, tolerability and efficacy of deoxycytidine and deoxythymidine as a therapeutic intervention. Additionally, another study (NCT06754098) is enrolling five adult participants with TK2 disease to assess the safety, tolerability and efficacy of oral deoxycytidine and deoxythymidine over a 24-month period. A combination of gene therapy with substrate enhancement has also proved to be a promising therapeutic strategy for TK2 deficiency in murine models [116].

Elamipretide is a tetrapeptide molecule that stabilizes cardiolipin of the inner mitochondrial membrane, leading to a reduction in toxic reactive oxygen species and thus improving mitochondrial function. A phase I/II trial including adult patients with PMM receiving intravenous elamipretide at the highest doses showcased that the use of elamipretide can improve exercise intolerance, specifically the 6 min walk test (6MWT) after five days of intravenous administration [117], and the authors highlighted the need of phase III trials for better assessment of the results in patients. Unfortunately, a subsequent phase III randomized placebo-controlled clinical trial assessing the efficacy and safety of elamipretide administered at a dose of 40 mg/d subcutaneously in PMM patients failed to demonstrate an improvement in the 6MWT. However, an intriguing observation from the study was that participants with PMM with nDNA defects, as opposed to those with mtDNA mutations, exhibited significantly better performance on the 6MWT [118].

Fibroblast growth factor 21 (FGF21) is a growth peptide hormone regulating lipid and glucose metabolism and thus energy homeostasis [119]. Although the expression of this hormone is not typical in skeletal muscle, it is highly expressed in mitochondrial myopathies where there are high levels of muscle stress [119]. Studies in deletor mice have shown that FGF21 acts as a circulating messenger regarding local and systemic stress responses in mitochondrial myopathies [70]. However, its potential as a therapeutic target still requires further investigation. In the same regard, growth differentiation factor 15 (GDF15) seems to be induced by the metabolic stress response, making it a possible candidate as a modulator of the metabolic features for metabolic and mitochondrial diseases [120]. A recent therapeutic approach utilizes photobiomodulation (PBM) for the treatment of diseases including dementia, Parkinson’s disease, stroke and even brain injury [121,122]. Study data show that near-infrared light (NIR) has the potency to stimulate the activity of cytochrome c oxidase (complex IV) of the mitochondrial respiratory chain, resulting in an increase in ATP synthesis and thus the enhancement of mitochondrial function [122]. Further studies need to be conducted in order to demonstrate whether the same mechanism can be used as a possible therapeutic approach for mitochondrial diseases, too.

Nicotinamide adenine dinucleotide (NAD (+)) precursors, like acipimox, can enhance mitochondrial function. In a randomized crossover trial with 21 type 2 diabetes patients, acipimox treatment improved mitochondrial respiration and upregulated mitochondrial gene expression, indicating a direct effect on skeletal muscle mitochondrial function [77].

Bezafibrate, a lipid-lowering agent commonly used to treat dyslipidemia, has also been investigated for its potential benefits in mitochondrial myopathies. In an open-label study of six patients with mitochondrial myopathy due to the m.3243A>G mutation, bezafibrate treatment for 12 weeks improved cardiac function and reduced complex IV-deficient muscle fibers without major adverse effects. However, it also increased mitochondrial disease biomarkers and disrupted metabolic profiles, highlighting potential concerns about long-term use [78].

Omaveloxolone, a nuclear factor (erythroid-derived 2)-like 2 (Nrf2) pathway activator recently approved for the treatment of Friedreich’s ataxia, has also been evaluated in mitochondrial myopathy. In a randomized, placebo-controlled trial, omaveloxolone up to 160 mg was well-tolerated in patients with mitochondrial myopathy but did not improve peak exercise capacity or 6MWT. However, the highest dose reduced heart rate and lactate levels during submaximal exercise, suggesting a potential improvement in mitochondrial efficiency and exercise tolerance [79].

Based on the promising results with CoQ10 and Idebenone, another para-benzoquinone compound, EPI-743, has been evaluated. In a phase 2A open-label trial, EPI-743 significantly improved neuromuscular function and quality of life in children with genetically confirmed Leigh syndrome, effectively halting disease progression without significant adverse effects [81]. In another small open-label trial, EPI-743 treatment in patients with LHON halted disease progression in four of five participants and reversed vision loss in most, with two achieving full recovery of visual acuity and no reported drug-related adverse events [82].

### 2.3. Emerging Therapeutic Approaches and Ongoing Clinical Trials

The mitochondrial unfolded protein response (mtUPR) is a critical cellular defense mechanism activated in response to mitochondrial dysfunction, and emerging therapeutic strategies aimed at modulating mtUPR hold promise for treating mitochondrial myopathies [123]. The mtUPR is a conserved stress-activated transcriptional response that promotes mitochondrial recovery by upregulating chaperones and proteases in response to the accumulation of misfolded proteins [123]. While this response may initially support mitochondrial function, prolonged mtUPR activation may contribute to mitochondrial damage and disease progression [123]. Over the past decade, various proteins and signaling pathways have been identified as potential therapeutic targets of the mtUPR [124]. Mitohormesis-based approaches, which mildly induce mitochondrial stress, could help selectively enhance beneficial aspects of the mtUPR [125]. Recent studies have highlighted the role of mtUPR activation in improving mitochondrial function in disorders such as MELAS and Leigh syndrome, with compounds like tetracyclines, pterostilbene, and mitochondrial cofactors showing potential therapeutic effects [126,127,128,129]. Moreover, treatments that increase NAD+ levels, such as NR and acipimox, which have been mentioned earlier, have been found to activate mtUPR pathways and improve mitochondrial function [129]. Together, these findings support the therapeutic potential of targeting the mtUPR to restore homeostasis in mitochondrial myopathies.

Recent advances in the treatment of mitochondrial myopathies have been strengthened by several ongoing clinical trials. These trials aim to explore innovative therapeutic approaches, including mitochondrial-targeted antioxidants, cell therapies and metabolic interventions. For instance, some trials are investigating dietary interventions and nutrient-based strategies, including NR (NCT05590468), glycerol tributyrate (NCT06792500) and the implementation of the ketogenic diet (NCT06013397), while others focus on restoring the nucleotide pool (NCT04802707, NCT06754098). Cell-based therapies, including the administration of autologous mesoangioblasts or autologous CD34+ hematopoietic stem and progenitor cells enriched with allogeneic placental-derived mitochondria (MNV-201), are also under investigation (NCT05962333, NCT06017869). Mitochondrial-targeted antioxidants like Sonlicromanol (NCT06451757), KL1333 (NCT05650229), Zagociguat (NCT06402123) and TTI-0102 (NCT06644534) are also being examined for their potential to enhance mitochondrial function. Additionally, PYC-001 is a small molecule drug that increases OPA1 protein levels, and its efficacy is currently being evaluated in adults with confirmed pathogenic mutation (NCT06461286). The outcomes of these studies will be instrumental in shaping the future of mitochondrial myopathy treatment and could offer new hope for patients with limited options. A summary of these ongoing clinical trials and their corresponding therapeutic approaches is provided in Table 2.

## 3. Management

**Figure 4:**
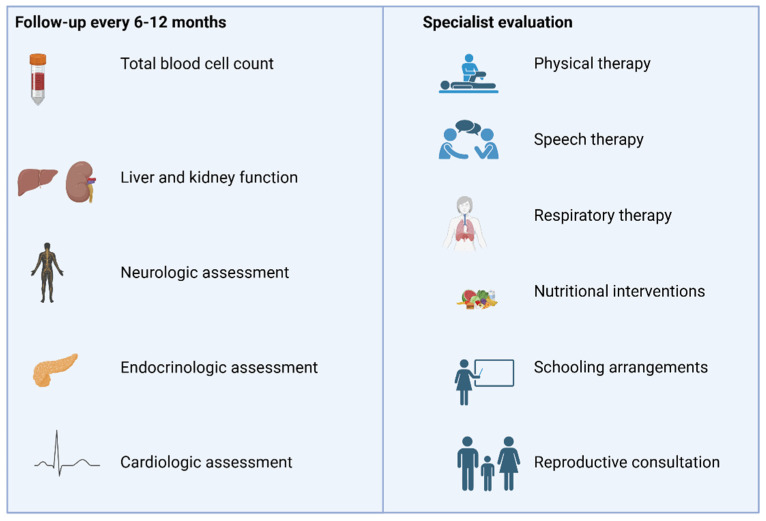
Management of patients with primary mitochondrial myopathies. Recommended follow-up includes regular assessments of hematologic, hepatic, renal, neurologic, endocrine and cardiac function, along with tailored evaluations by specialists in nutrition, physical and respiratory care and genetic counseling, depending on the patient’s clinical presentation. The figure was prepared using the BioRender platform (under license to DPB, Created in BioRender. Bogdanos, D. (2025) https://BioRender.com/bbmz83a).

Regarding the management of mitochondrial myopathies, there is a need for a multidisciplinary approach, the focus of which is many times the prevention of complications and treatment of disabilities (Figure 4).

To date, no studies have shown hard evidence concerning recommendations about the follow-up of these patients, and most of the available guidelines are based upon experts’ opinions [130]. Most guidelines suggest a follow-up every 6–12 months, which should include a total blood cell count, liver and kidney function examination and neurologic, endocrinologic (screen for diabetes) and cardiologic (electro- and echocardiogram tests, especially in patients with syndromes known for cardiac conduction defects) assessment. Adjustments of this initial follow-up should be made according to the specific underlying mitochondrial disease. Evaluation from other specialists like ophthalmologists, gastroenterologists, nephrologists, pulmonologists and immunologists should be sought depending on the clinical manifestations of each patient [130,131].

Infections in patients with mitochondrial diseases are linked to serious complications, and, as a result, immunization for these patients is strongly encouraged by experts [19].

As far as medications are concerned, the use of specific medications such as valproic acid, metformin, aminoglycosides and linezolid is advised to be avoided, due to the risk of mitochondrial toxicity [130,132].

Mitochondrial myopathies can lead to limited muscle functionality, strength and stamina. The aim of physical therapy in these patients should be to maintain muscle function and to improve balance, gait and endurance [133]. Exercise should be personalized for each patient, although most physiotherapy approaches start with short and low-intensity exercises, escalating according to the patient’s status and progression [134].

Speech therapy can help not only in speech and communication disorders but also with swallowing problems and dysphagia, preventing respiratory problems like aspiration pneumonia [134,135].

Mitochondrial myopathies may affect respiratory muscles too, even resulting in respiratory failure. Respiratory therapy can help in more efficiently managing secretions and enhancing respiratory capacities. However, in severe and advanced forms of mitochondrial disease, treatment with oxygen supplementation or even ventilation support may be needed [136].

Evaluation of each patient’s nutritional status should be made by a nutritionist, and intervention with dietary strategies should be implemented in order to meet every patient’s nutritional needs and to battle nutritional deficiencies. Potential interventions also include enteral feeding or even parenteral nutrition [136,137].

Children with mitochondrial myopathies may face problems attending school, showcasing the need for specialized school arrangements and potentially home tutoring.

Patients with diagnosed mutations to nDNA or mtDNA can seek consultation with a geneticist for matters regarding reproduction and family programming. Reproductive options include either sperm or oocyte donation, as well as a more modern method of mitochondrial donation, which has been approved by law regulations for clinical use in UK since 2015 [138].

## 4. Conclusions

Mitochondrial myopathies are genetic disorders, impairing mitochondrial functions, with a great variety of clinical syndromes and manifestations. Management of these disorders requires a multidisciplinary approach and the collaboration of many specialties, as they can affect different and multiple organs, especially those rich in mitochondria, such as muscles and the heart. As of now, no approved treatment for mitochondrial disease exists, and treatment strategies aim at symptomatic relief. Most guidelines are based upon experts’ opinions, and treatment options focus mainly on supplementation with vitamins and cofactors, for which hard evidence of benefit is still lacking. Nonetheless, the therapeutic landscape is expanding beyond symptomatic management, with promising disease-modifying approaches emerging from preclinical and early clinical studies. Further clinical trials for the already applied treatment options as well as novel experimental therapies are of utmost importance in order to improve patients’ quality of life.

## Figures and Tables

**Table 1:** Possible treatment options for primary mitochondrial myopathies.

Substance	Doses	Clinical Outcomes	Reference
Ubiquinol	In PMM with Q10 deficiency adults: p.o. 5–600 mg/daily pediatric: p.o. 2–8 mg/kg/daily divided in 2 doses	Amelioration of symptoms in syndromes characterized by Q10 deficiency, antioxidant and pro-oxidant role	[55,58]
Alpha lipoic acid	50–200 mg/daily	Antioxidant role	[55]
Rifoflavin (B2)	p.o. 50–400 mg/daily	Amelioration of symptoms, slowing of disease progression	[60]
Nicotinamide riboside (B3)	p.o. 400 mg/kg/daily	Delay of early- and late-stage disease progression	[69]
Carnitine	adults: 3 g/daily in 3 doses pediatric: 100 mg/kg/daily in 3 doses	Restoration of free carnitine levels and removal of accumulating toxic acyl compounds	[70]
Creatinine	adults: 10 g p.o. daily divided 2–3 times pediatric: 0.1g/kg p.o. daily	Increase in high-intensity, isometric, anaerobic and aerobic power	[60]
L-arginine	Acute (1–3 days): iv 500 mg/kg/daily Maintenance: p.o. or iv 150–300 mg/kg/daily in 3 doses	Reduction in the severity and frequency of metabolic strokes in patients with MELAS and other forms of mitochondrial disease	[55,71]
Elamipretide	iv 0.25 mg/kg/h over 2 h for 5 consecutive days	Increase in exercise performance, improvement in the distance walked on the 6MWT	[72]
Deoxynucleoside monophosphates, deoxynucleosides	300–400 mg/kg/daily	Halt in early-onset disease progression, amelioration of muscle weakness, reversion of early onset tetraplegia, improvements in mechanical ventilation	[73]
Idebenone	900 mg/day divided into three doses	Improvement in visual acuity, protection from loss of color vision	[74,75,76]
Acipimox	250 mg three times daily for 2 weeks	Enhanced mitochondrial respiration, increased expression of mitochondrial genes	[77]
Bezafibrate	600–1200 mg daily for 12 weeks	Improved cardiac function, reduced proportion of complex IV-deficient muscle fibers	[78]
Omaveloxolone	Up to 160 mg for 12 weeks	Reduced heart rate and lowered plasma lactate levels during submaximal exercise	[79]
Taurine	9 g or 12 g per day for 52 weeks	Reduced frequency of stroke-like episodes in MELAS patients	[80]
EPI-743	100 mg three times daily	Improved neuromuscular function in children with Leigh syndrome	[81]
	100–400 mg three times daily	Reversed vision loss in most patients with LHON	[82]

MELAS: mitochondrial encephalomyopathy lactic acidosis and stroke-like episodes, LHON: Leber’s hereditary optic neuropathy, 6MWT: six-minute walk test.

**Table 2:** Ongoing clinical trials investigating therapeutic approaches for mitochondrial myopathies.

Intervention	Phase Study	Participants	ClinicalTrials.gov ID
Nicotinamide riboside (B3)	II	Adults with mitochondrial myopathy	NCT05590468
Deoxycytidine and deoxythymidine	II	Pediatric patients with mtDNA depletion syndromes	NCT04802707
Deoxycytidine and deoxythymidine	II	Adults with TK2 disease	NCT06754098
Autologous mesoangioblasts	II	Adults with m.3243A>G mutation	NCT05962333
MNV-201	I	Pediatric patients with Pearson syndrome	NCT06017869
KL1333	II	Adult patients with primary mitochondrial disease	NCT05650229
Sonlicromanol	III	Adults with a genetically confirmed mtDNA tRNALeu(UUR) m.3243A>G Variant	NCT06451757
Zagociguat	II	Adults with MELAS syndrome	NCT06402123
TTI-0102	II	Patients > 16 years old with MELAS syndrome	NCT06644534
Ketogenic Diet	ΝA	Patients with MELAS syndrome	NCT06013397
Glycerol tributyrate	I	Adults with MELAS or LHON-Plus	NCT06792500
PYC-001	Ι	Adults with confirmed OPA1 mutation	NCT06461286

MELAS: mitochondrial encephalomyopathy lactic acidosis and stroke-like episodes, mtDNA: mitochondrial DNA, NA: not applicable, LHON: Leber’s hereditary optic neuropathy, TK2: thymidine kinase 2.
